# Sociodemographic characteristics associated with oral health literacy in adults: A cross-sectional study

**DOI:** 10.21142/2523-2754-1304-2025-263

**Published:** 2025-11-08

**Authors:** Sergio Orozco-Vásquez, Teresa Evaristo-Chiyong

**Affiliations:** 1 SAETA Research group. School of Dentistry, Universidad Nacional Mayor de San Marcos. Lima, Peru. 12050077@unmsm.edu.pe tevaristoc@unmsm.edu.pe Universidad Nacional Mayor de San Marcos SAETA Research group School of Dentistry Universidad Nacional Mayor de San Marcos Lima Peru 12050077@unmsm.edu.pe tevaristoc@unmsm.edu.pe

**Keywords:** health literacy, oral health, social determinants of health, adult, alfabetización en salud, salud bucal, determinantes sociales de la salud, adulto

## Abstract

**Objective:**

To determine the sociodemographic characteristics associated with oral health literacy (OHL) in Peruvian adults attending a public health center in Lima.

**Materials and Methods:**

This cross-sectional study included 384 Peruvian adults aged 19 to 64 who visited a health center in Metropolitan Lima. OHL was measured using the Peruvian version of the Health Literacy in Dentistry-14 (HeLD-14) score. Sociodemographic characteristics, including gender, age, place of residence, number of children, health insurance status, educational level, occupation, and monthly income, were collected. Bivariate analysis was performed using the nonparametric Mann-Whitney U, Kruskal-Wallis, and Spearman correlation tests, while multivariate analysis was conducted using the linear regression model with a significance level of 5%.

**Results:**

The average OHL score was 48.60 ± 6.89. Literacy was higher in women (p = 0.004) and among those with higher incomes (p = 0.002). Conversely, literacy decreased with increasing age (p = 0.022) and number of children (p = 0.033). In the adjusted model, an association was found with being female (β = 0.097, p = 0.001), younger age (β = -0.003, p = 0.015), and a monthly income greater than five minimum wages (β = 0.100, p = 0.025).

**Conclusion:**

OHL in an adult population in an urban area tends to be high and is associated with sociodemographic characteristics such as gender, age, and monthly income.

## RESUMEN

**Objetivo:**

Determinar las características sociodemográficas asociadas a la alfabetización en salud oral en adultos peruanos que acuden a un centro de salud público de Lima.

**Materiales y métodos:**

Estudio transversal en 384 peruanos adultos con edades comprendidas entre 19 y 64 años que acudieron a un centro de salud de Lima Metropolitana. La alfabetización en salud oral se midió a través del puntaje obtenido a través del Health Literacy in Dentistry-14 (HeLD-14), versión peruana, y se recopilaron características sociodemográficas como sexo, edad, lugar de residencia, número de hijos, tenencia de seguro de salud, grado de instrucción, ocupación e ingreso mensual. El análisis bivariado se realizó a través de las pruebas no paramétricas U de Mann-Whitney, Kruskal-Wallis y correlación de Spearman; y el análisis multivariado, a través del modelo de regresión lineal considerando un nivel de significancia del 5%.

**Resultados:**

El promedio de alfabetización en salud oral fue de 48,60 ± 6,89. La alfabetización fue mayor en mujeres (p = 0,004), así como en los que declararon ingresos mayores (p = 0,002). A mayor edad (p = 0,022) y mayor número de hijos (p = 0,033) la alfabetización fue menor. En el modelo ajustado, se encontró asociación entre ser de sexo femenino (β = 0,097 p = 0,001), menor edad (β = -0,003 p = 0,015) e ingreso mensual mayor a cinco sueldos mínimos (β = 0,100 p = 0,025).

**Conclusión:**

La alfabetización en salud oral en una población adulta en una zona urbana tiende a ser alta y está asociada con características sociodemográficas como sexo, edad e ingreso mensual.

## INTRODUCTION

Research has shown that oral diseases, such as caries, periodontal disease, and oral cancer, may be a consequence of disparities in the sociodemographic characteristics of the population ^1^^,^^2^. These characteristics are considered fundamental determinants of health behaviors and outcomes ^3^. 

Oral health literacy (OHL) refers to a person’s ability to obtain, process, and understand information about basic services that enable them to make informed decisions about their oral health ^4^^,^^5^.

Poor OHL is linked to adverse oral health outcomes, including dental caries, tooth loss, and periodontal disease ^6^^,^^7^, which in turn affect oral health-related quality of life ^8^. 

Various instruments exist for measuring OHL, some based on reading ability and numeracy skills, such as the Test of Functional Health Literacy in Dentistry (ToFHLiD), or on word recognition, such as the Rapid Estimate of Adult Literacy in Dentistry (REALD) ^9^^,^^10^. The Health Literacy in Dentistry (HeLD) assesses OHL in a multidimensional manner by considering seven domains: communication, comprehension, receptivity, utilization, support, economic barriers, and access, allowing for a more comprehensive and functional assessment of this construct ^11^. It has a short version, HeLD-14, which was originally validated in English for an Australian adult population ^12^. It has been adapted and validated into Brazilian Portuguese ^13^, Mandarin Chinese ^14^, Spanish in Colombia ^15^, and Spanish in Peru ^16^, all demonstrating good psychometric properties.

Health literacy can be influenced by the sociodemographic context of the population ^17^^,^^18^, with characteristics such as educational level, occupation, and income being associated with it ^19^^-^^21^, although there is no consensus on this issue. Scientific evidence is scarce in the context of Latin American countries, such as Peru, where access to health and education services is conditioned by socioeconomic and geographic status.

Understanding the level of literacy in adults and how it may be affected by social characteristics will facilitate the establishment of differentiated health education and promotion strategies that contribute to reducing gaps in oral health inequality ^22^. In this context, the objective of the research was to determine the sociodemographic characteristics associated with OHL in Peruvian adults attending a public health center in Lima.

## MATERIALS AND METHODS

### Ethical aspects

This cross-sectional research was approved by the Institutional Ethics Committee of the Faculty of Dentistry of the National University of San Marcos (No. 009-CEI-FO-2023). All participants signed an informed consent form prior to participation.

### Population and sample

The population consisted of Peruvian adults of both genders between the ages of 19 and 64, recruited at the San Isidro Health Center of the Ministry of Health in Metropolitan Lima in 2023. Inclusion criteria required participants to have received dental care or to have accompanied a patient and received instructions from the dentist in the last 10 years. Those who did not sign the informed consent form or who could not complete the questionnaire independently were excluded. The sample size was calculated with a confidence level of 95%, a standard deviation of 8.90, and a margin of error of 0.89, based on data from a pilot study, resulting in a required sample size of 384. Selection was performed consecutively until the required sample size was achieved.

### Oral health literacy variable

This was measured using the Peruvian version of the HeLD-14 instrument.^16^ The internal consistency of the instrument was evaluated in a pilot sample, yielding a Cronbach’s alpha of 0.887 and McDonald’s omega of 0.894.

This questionnaire consists of 14 questions divided into seven conceptual domains: receptivity, understanding, support, economic barriers, access, communication, and utilization. The response options were evaluated using a Likert scale from 0 to 4 according to the difficulty of performing each task, with 0 indicating “cannot” and 4 indicating “no difficulty.” The total score could range from 0 to 56 points, with higher scores indicating higher literacy ^16^.

### Sociodemographic characteristics variables

Gender, age, place of residence, number of children, health insurance coverage, level of education, occupation, and monthly income were analyzed based on the minimum wage in Peru (approximately USD 280).

The questionnaire was administered in the waiting room of the health center, either before or after care, by a trained interviewer who was not affiliated with the health center. The approximate time to complete the questionnaire was 20 minutes. Only one attempt was made to contact each participant. To minimize response bias, including social desirability bias, the questionnaire was coded to maintain anonymity during data collection and analysis.

### Statistical analysis

Relative and absolute frequency tables were used to describe qualitative variables, while measures of central tendency and dispersion were employed for quantitative variables. Bivariate inferential analysis was performed using Spearman’s correlation, Kruskal-Wallis, and Mann-Whitney U tests, as the OHL variable was not normally distributed. For the multivariate analysis, a linear regression model was used, with logarithmic transformation to meet its assumptions. A significance level of 5% was considered. The data were processed using IBM Statistics SPSS 26.0 statistical software.

## RESULTS

A total of 384 adults aged between 19 and 64 years were surveyed. The average oral health literacy (OHL) score was 48.60 (± 6.89) points. The majority of participants were female (66.7%, n = 256), with an average age of 33.61 (± 11.04) years, residing primarily in Lima Centro (72.4%, n = 278), and having an average of 0.73 (± 1.11) children. Seventy-three point seven percent (n = 283) had state health insurance, while 81.0% (n = 311) had higher education, and 54.7% (n = 210) were employed or workers. Additionally, 41.9% (n = 161) reported a monthly income of up to one minimum wage (Table 1).

**Table 1:** Sociodemographic characteristics of adults who attended a Health Center in Lima. (n = 384)

Sociodemographic characteristics	n	%
Sex		
Male	128	33.3
Female	256	66.7
Age (years)	33.61 ± 11.04*		
Place of residence		
Central Lima districts	278	72.4
Other districts	106	27.6
Number of children	0.73 ± 1.11*		
Health insurance		
No	26	6.8
State insurance (SIS/EsSalud)	283	73.7
Private insurance	75	19.5
Education level		
Primary	5	1.3
Secondary	68	17.7
Higher	311	81.0
Occupation		
Employer-Self-employed worker	113	29.4
Employee-Laborer	210	54.7
Unpaid (Domestic worker, Unpaid family worker, and Unemployed)	61	15.9
Economic income		
Up to one MW	161	41.9
More than one MW to three MW	128	33.3
More than three MW to five MW	47	12.2
More than five MW	48	12.5

*=Mean ± standard deviation. MW=Minimum wage.

Women had a higher literacy score of 49.36 (± 6.43; p = 0.004), as did those who were employed (49.38 ± 6.51; p = 0.043). A relationship was found with monthly income (p = 0.002), indicating that higher income corresponded to higher literacy, as well as with age (p = 0.022) and number of children (p = 0.033), where lower age and fewer children corresponded to higher literacy. No statistical significance was found regarding educational level, place of residence, or health insurance coverage (p > 0.05; Table 2).

**Table 2:** Oral health literacy according to sociodemographic characteristics of adults who attended a Health Center in Lima. (n = 384)

Sociodemographic characteristics	Oral health literacy (HeLD-14)				
	Mean± SD.	Me	Min.	Max.	p
Sex					
Male	47.10 ± 7.54	49.0	22	56	0.004*
Female	49.36 ± 6.43	51.0	16	56	
Place of residence					
Central Lima districts	48.54 ± 6.89	50.0	16	56	0.614*
Other districts	48.78 ± 6.93	50.0	28	56	
Health insurance					
No	49.65 ± 5.80	52.0	37	56	0.096†
State insurance (SIS/EsSalud)	48.16 ± 7.13	50.0	16	56	
Private insurance	49.92 ± 6.16	52.0	30	56	
Education level					
Primary	47.20 ± 9.28	47.0	33	56	0.058‡
Secondary	46.69 ± 8.60	49.5	16	56	rho = 0.097
Higher	49.05 ± 6.37	50.0	22	56	
Occupation					
Employer-Self-employed worker	47.68 ± 7.18^a^	49.0	22	56	0.043†
Employee-Laborer	49.38 ± 6.51^b^	51.0	16	56	
Unpaid (Domestic worker, Unpaid family worker, and Unemployed)	47.64 ± 7.39^ab^	50.0	23	56	
Economic income					
Up to one MW	47.65 ± 7.40	50.0	16	56	
More than one MW to three MW	48.66 ± 6.18	50.0	27	56	0.002‡
More than three MW to five MW	50.17 ± 6.50	53.0	31	56	rho = 0.157
More than five MW	50.13 ± 6.94	52.0	22	56	
Age (years)	rho = - 0.117; p = 0.022‡				
Number of children	rho = - 0.109; p = 0.033‡				

Me=Median, MW=Minimum wage *: Mann-Whitney U †: Kruskal-Wallis, different letters indicate differences between groups (Dunn-Bonferroni post hoc analysis) ‡: Spearman correlation.

The multivariate model was adjusted based on the variables that were significant in the crude model. The literacy score increased by 0.097 (Beta = 0.097, p = 0.001) for females and by 0.100 (Beta = 0.100; p = 0.025) for those with a monthly income greater than five minimum wages. Conversely, with increasing age, the literacy score decreased by 0.003 (Beta = -0.003; p = 0.015; Table 3).

**Table 3:** Crude and adjusted multivariate analysis between sociodemographic characteristics and oral health literacy.(n = 384)

Sociodemographic characteristics	Crude			Adjusted		
	Beta	CI 95% (ß)	p	Beta	CI 95% (ß)	p
Sex						
Female (ref Male)	0.090	0.032 - 0.148	0.002*	0.097	0.039 - 0.155	0.001*
Place of residence						
Central Lima districts (ref Other)	- 0.014	- 0.076 - 0.047	0.646		------------	
Education level						
Secondary (ref Primary)	- 0.087	- 0.159 - -0.015)	0.019*	- 0.053	- 0.298 - 0.191	0.669
Higher (ref Primary)	0.084	0,014 - 0,154	0.019*	- 0.001	- 0.241 - 0.239	0.994
Occupation						
Employer-Self-employed worker (ref Unpaid)	- 0.055	- 0.115 - 0.006	0.076		----------------	
Employee-Laborer (ref Unpaid)	0.073	0.018 - 0.128	0.009*	0.035	- 0.023 - 0.093	0.241
Economic income						
More than one MW to three MW(ref < 1 MW)	- 0.011	- 0.070 - 0.047	0.703		----------------	
More than three MW to five MW (ref < 1 MW)	0.089	0.005 - 0.173	0.039*	0.069	- 0.018 - 0.157	0.121
More than five MW (ref < 1 MW)	0.090	0.007 - 0.173	0.034*	0.100	0.013 - 0.187	0.025*
Health insurance						
State insurance (ref No)	- 0.068	- 0.131 - -0.006)	0.032*	- 0.047	- 0.150 - 0.061	0.393
Private insurance (ref No)	0.072	0,003 - 0.142	0.041*	- 0.014	- 0.135 - 0.107	0.821
Age	- 0.003	- 0.006 - (- 0,001)	0.009*	- 0.003	- 0.005 - -0.001)	0.015*
Number of children	- 0.025	- 0.050 - 0.000	0.052	-------		

*:Significant. The model was adjusted to the variables that were significant. F = 3.939 p < 0.001 Adjusted R2 = 0.065. MW = Minimum wage

## DISCUSSION

The research revealed that adults’ oral health literacy (OHL) had high average scores. Previous studies ^18^^-^^19^^,^^23^ conducted on adults attending public health centers in urban areas and using the HeLD-14 instrument reported values below the average found in this study.

When evaluated according to sociodemographic characteristics, a difference was observed based on gender, with females achieving higher average scores. This finding aligns with Mahmud’s ^20^ research, which indicated that younger women with higher levels of education had higher literacy scores. However, Henderson ^19^, Noor ^24^, and Tam ^25^ found no gender differences. In the present study, the number of females was twice that of men, and females tended to be more informed about health issues, as they are primarily responsible for the care of their children, which may explain the observed results.

Younger participants exhibited higher OHL. Similar findings were reported in a study where individuals under 40 years of age had higher literacy compared to those aged 40 to 60 ^20^. In contrast, Noor ^24^^)^ found that older individuals had higher OHL. Assunção ^18^, Henderson ^19^, and Tam ^25^^)^ found no relationship between these variables. Most participants in the present study were younger than those in previous studies (under 40 years of age). This age group is often more connected to various social networks and online information platforms, which may facilitate greater access to oral health information, resulting in higher literacy.

No differences were found in OHL averages across different districts of Lima. This study primarily evaluated individuals from urban areas, suggesting that the situation in rural areas, with their distinct social and demographic contexts, may differ significantly from the findings of this research, as indicated by previous studies ^26^^)^ comparing health literacy skills in urban and rural populations.

The number of children among participants, as well as their age, exhibited an inverse relationship with OHL; thus, fewer children correlated with higher literacy levels. No previous studies corroborate this result, indicating a need for further research. This finding may relate to the participants’ age, as those with more than one child tended to be over 40 years old, and older participants generally had lower literacy levels.

The literacy score did not differ according to the types of health insurance; however, in the crude regression analysis, using the lack of health insurance as a reference, it was observed that having private insurance increased the literacy score by 0.072, while having state health insurance decreased it by 0.068. This raises questions about whether the procedures and communication methods employed in state public health institutions negatively affect decision-making capacity. This result aligns with a study indicating that individuals affiliated with state insurance had poor health literacy, which may be attributed to educational and economic disparities ^27^.

Literacy was not related to educational level in either the bivariate analysis or the adjusted multivariate model. However, in the crude model, when using no educational level as a reference, it was observed that higher educational level increased the literacy score by 0.084. These discrepancies may be attributed to differences in the mathematical calculations of the tests used. Other authors ^27^^-^^28^_^,30)^_ have found a relationship between educational level. In the present study, over 80% of respondents had higher education, either technical or university studies, with only five cases of primary education, and the overall literacy score in the entire sample tended to be high. Additionally, the population came from an urban area with consistent access to information and services, which, regardless of educational level, could contribute to adequate literacy. 

Significant differences were found in the occupation variable, with employees or workers achieving the highest OHL scores. Henderson ^19^, Mahmud ^20^, and Dieng ^28^^)^ also found differences according to occupation. 

Higher monthly income was associated with higher OHL. Previous studies also demonstrate a strong direct relationship between these two variables ^18^^-^^20^^,^^24^. Earning a monthly income above the Peruvian minimum wage reflects other favorable sociodemographic characteristics, such as a high level of education and access to better sources of information and health services, thereby facilitating the development of greater OHL.

After analyzing all sociodemographic variables collectively, it was found that high OHL is associated with being female, younger age, and a monthly income greater than five times the minimum wage in Peru. Although the beta coefficients may be considered low at the individual level, in public health, even a minimal increase can significantly impact the population. Other studies have identified additional associations, including marital status ^18^^)^ and ethnicity ^25^^,^^29^.

## LIMITATIONS

Among the limitations of this study, it is important to note that the population belonged to a public health center located in an urban district of Lima and had access to health care, as the questionnaire required prior experience with dental care. Therefore, the generalization of the results should be applied to similar contexts. The study design was cross-sectional, preventing the establishment of causal relationships between variables. The questionnaire used represents one of several theoretical constructs available for measuring OHL, which may explain the differences found in the literature. The strength of this study lies in its evaluation of literacy using an instrument whose theoretical construct is multidimensional in nature and adapted to the Peruvian population. Future research is recommended in rural populations and indigenous communities, whose geographical, cultural, and socioeconomic contexts may differ and impact literacy levels and decision-making in oral health.

## CONCLUSION

Oral health literacy in an adult population in an urban area tends to be high and is associated with sociodemographic characteristics such as being female, having a monthly income above five minimum wages, and being younger.

## Source(s) of support:

Self-financed
